# The prognostic efficacy of the 8th edition UICC TNM classifications for gastric cancer in Chinese patients: A study based on follow-up system of nursing department

**DOI:** 10.1097/MD.0000000000012284

**Published:** 2018-09-07

**Authors:** Liang Liu, Yu Bai, Huizi Gu, Haitao Zhu, Ying Yu, Ping Lu, Yuxi Wang, Hao Zhang, Min Li

**Affiliations:** a Emergency Intensive Care Unit; b Department of Thoracic Surgery; c Department of Internal Neurology, the Second Hospital of Dalian Medical University, Dalian; d Department of Gastric Surgery, Liaoning Cancer Hospital and Institute; e Liaoning Medical Device Test Institute; f Department of Surgical Oncology, the First Hospital of China Medical University, Shenyang; g Department of General Surgery; h Department of Nursing, the Second Hospital of Dalian Medical University, Dalian, China.

**Keywords:** Chinese population, gastric cancer, tumor-node-metastasis staging

## Abstract

This study aimed to analyze the applicability of the Union for International Cancer Control (UICC) tumor-node-metastasis (TNM) classification 8th edition for Chinese patients with gastric cancer.

A review of all inpatient and outpatient records of patients with gastric cancer was conducted in the First Affiliated Hospital of China Medical University and Liaoning Cancer Hospital and Institute. All patients who met the inclusion criteria and were seen from January 1980 through December 2009 were included in the study. The primary outcome was 5-year survival, which was analyzed according to the decade of diagnosis and TNM classifications.

Two thousand five hundred fifty-four patients were enrolled in this study. When classified according to the UICC TNM classification of gastric cancer 8th edition, the prognoses of patients with stage IIIB (n = 250) and stage IIIC (n = 101) disease were not significantly different (*P* = .332). However, if T4aN2 patients were classified as having stage IIIB disease, and T4bN2 and T4aN3a patients were classified as having stage IIIC disease, the prognoses of stage IIIB (n = 221) and stage IIIC (n = 172) patients were significantly different (*P* = .03).

Classifying T4bN0 patients as having stage IIIB disease, and T4bN2 and T4aN3a patients as having stage IIIC disease according to the 8th edition of UICC gastric cancer TNM classifications better stratified Chinese patients and predicted prognoses.

## 1 Introduction

In the past few decades, the survival of patients with gastric cancer was significantly prolonged. Particularly in the last 10 years, remarkable improvements have been made in the comprehensive treatment of gastric cancer. With the gradual standardization of treatment and reduction of surgical complications, the treatment of early gastric cancer is becoming increasingly standardized, and a systematic, comprehensive treatment regimen has gradually been developed. Outcomes for patients with gastric cancer have improved because of early diagnosis, radical surgery, and the development of adjuvant therapy. However, late-stage gastric cancer patients still have poor prognoses.^[1–3]^ Treatment of these patients is still controversial. We believe that the disagreement regarding treatment arises because the classification standards applicable to late-stage gastric cancer patients still require further refinement. Only relatively refined classification standards can provide valuable information for patient treatment and prognosis.

We previously conducted a retrospective analysis of the survival of Chinese gastric cancer patients with different stages of disease between 1980 and 2003 according to the 6th edition Union for International Cancer Control (UICC) tumor-node-metastasis (TNM) gastric cancer classification.^[4]^ Later, we also conducted a new statistical analysis according to the 7th edition classification standards released in 2010^[5]^ and analyzed the applicability of the 7th edition UICC TNM gastric cancer classification for Chinese patients; we proposed a classification method improvement applicable for late-stage Chinese gastric cancer patients.^[6]^ In early 2017, the UICC released the 8th edition classification standards, and we also analyzed the applicability of the standards for Chinese patients according to the clinical pathology and follow-up data of Chinese patients. The aim is to compare the new and old standards, analyze their advantages and disadvantages, and understand the reasons for these advantages and disadvantages to aid future clinical testing and the proposal of new classifications.

With regard to the 2 studies describe above, we collected complete 5-year follow-up data for a portion of patients in chronological order after 2000, systematically performed a retrospective review of their clinical pathology data and follow-up data, and reclassified TNM stages for all patients according to the 8th edition.

This study aimed to conduct a systematic and comprehensive review of Chinese gastric cancer patients to investigate whether the 8th edition TNM classification standards are applicable to Chinese gastric cancer patients, in particular late-stage gastric cancer patients. We also aimed to provide a reference for more accurate staging and effective treatment in the future.

## 2 Methods

### 2.1 Patients

We enrolled 2554 patients with histologically confirmed gastric cancer who underwent surgery at the First Hospital of China Medical University and the Liaoning Cancer Hospital and Institute between 1980 and 2011. All patients had histologically confirmed gastric cancer, underwent surgery, and had complete medical records available.

All patients were followed up by telephone interviews through the follow-up system of nursing department. The last follow-up was in July 2017. Clinical, surgical, and pathological findings, and all follow-up data were collected and recorded in the database of nursing department. The study protocol was approved by the Ethics Committee of the Second Hospital of Dalian Medical University, the First Hospital of China Medical University and the Liaoning Cancer Hospital and Institute.

### 2.2 Endpoints and follow-up

The primary endpoint was 5-year survival. Overall survival was calculated from the date of surgery until death or to the last follow-up contact. Data for a patient were censored at the last follow-up when they were alive. Follow-up assessments were conducted every 6 months for the first 5 years after surgery, and every 12 months thereafter until death.

### 2.3 Ethics approval and consent to participate

The study was given ethical approval with Ethical Committee of the Second Hospital of Dalian Medical University, the First Hospital of China Medical University and the Liaoning Cancer Hospital and Institute and all the patients had given written informed consent.

### 2.4 Statistical analysis

Kaplan–Meier survival curves were used to estimate patient survival. Cox proportional hazards regression models were used to assess the associations of risk factors with survival. For univariate analyses, the prognostic factor of interest and the diagnosis period were covariates in the Cox regression model. Two-sided *P* values were calculated for all tests and are reported here. *P* values less than .05 were considered statistically significant. Analyses were performed using SPSS software, version 23.0.

## 3 Results

### 3.1 Patient characteristics

Two thousand five hundred fifty-four patients were enrolled in this study. Patient characteristics are shown in Table 1. The median patient age was 58 years, and the majority of patients were male. The median tumor size was 5 cm, and over half were located in the antrum. Nearly half of the tumors were classified with a T stage of T2, and the largest proportion had an N stage of N0. Over 60% of the tumors had a gross type of Borrmann III. Nearly half of the patients underwent radical resection, and the majority of patients underwent subtotal gastrectomy and D2 lymph node dissection. The proportion of patients receiving adjunctive therapy was 20%.

**Table 1:**
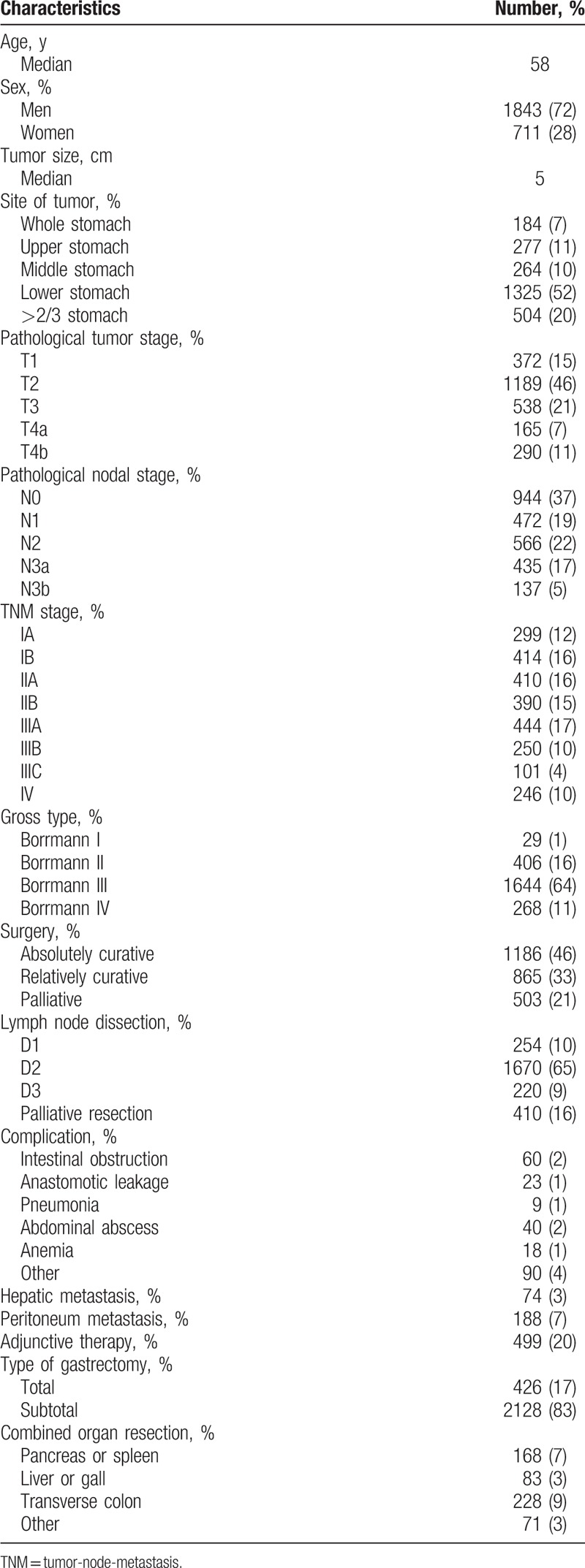
Characteristics of population from the 3 periods (n = 2554).

Univariable analysis revealed that as the T stage and N stage gradually increased, HR also increased, showing that the overall condition of the data was ideal. Multivariate Cox proportional hazards models for gastric cancer are shown in Table 2 . In the Cox model for gastric cancer, after adjusting for 13 variables, there were significant associations between tumor site, gross appearance, T stage, N stage, type of surgery, joint organ removal, hepatic metastasis, peritoneum metastasis, and adjunctive therapy and patient survival.

**Table 2:**
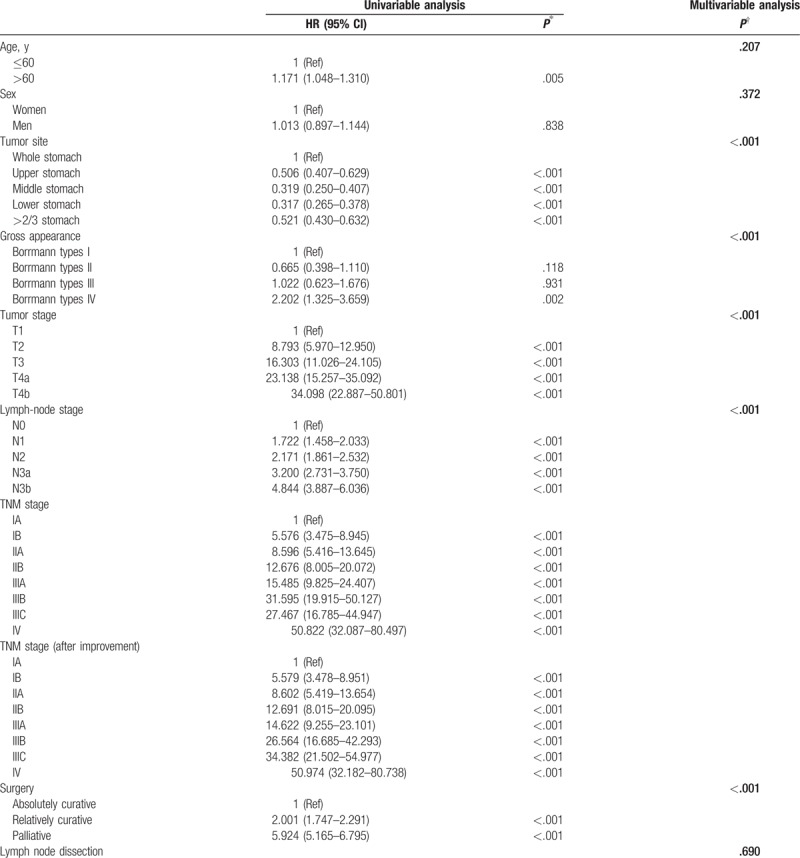
Hazard ratio (HR) for death in population (n = 2554)—univariable and multivariable analyses.

**Table 2 (Continued):**
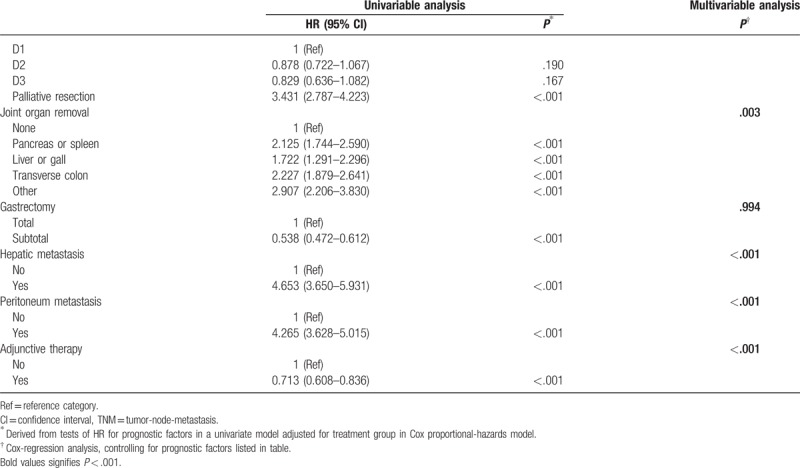
Hazard ratio (HR) for death in population (n = 2554)—univariable and multivariable analyses.

### 3.2 Improvement to the 7th edition UICC classification

**Figure 1:**
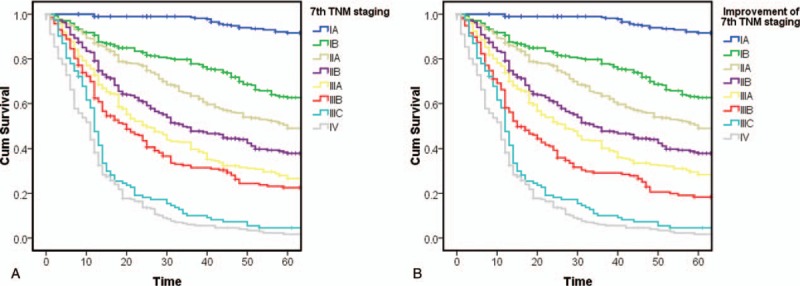
(A) Kaplan–Meier survival curves for patients according to the 7th edition Union for International Cancer Control (UICC) classification; (B) Kaplan–Meier survival curves for patients after classifying T4bN0 as stage IIIA disease in the 7th edition UICC classification.

In previous studies, we found that according to the 7th edition classification standards, there was no significant difference between stages IIIA and IIIB (Fig. 1A). Thus, we proposed an improvement to the 7th edition TNM classification, namely, moving stage T4bN0 from stage IIIB to stage IIIA (Fig. 1B).

### 3.3 Improvements to the 8th edition UICC classification

**Figure 2:**
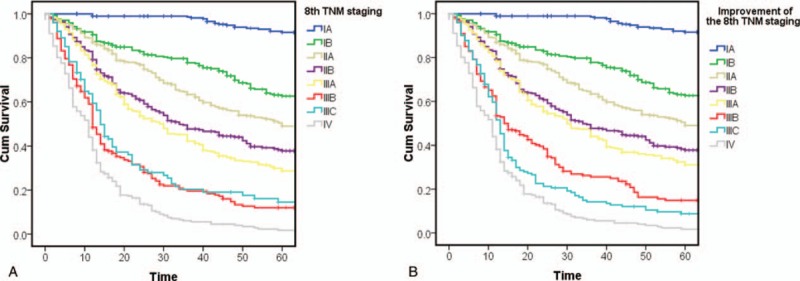
(A) Kaplan–Meier survival curves for patients according to the 8th edition Union for International Cancer Control (UICC) classification; (B) Kaplan–Meier survival curves for patients after improvement in the 8th edition UICC classification.

However, after the 8th edition standards were released, we found that, according to the 8th edition TNM classification standards, the difference in survival time between stage IIIB (n = 250) and stage IIIC (n = 101) disease was not statistically significant (*P* = .332) (Fig. 2A).

**Figure 3:**
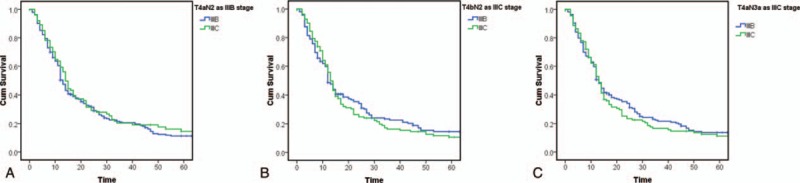
(A) Kaplan–Meier survival curves for patients after classifying T4aN2 as stage IIIB disease in the 8th edition Union for International Cancer Control (UICC) classification; (B) Kaplan–Meier survival curves for patients after classifying T4bN2 as stage IIIC disease in the 8th edition UICC classification; (C) Kaplan–Meier survival curves for patients after classifying T4aN3 as stage IIIC in the 8th edition UICC classification.

The 7th staging standards were used to classify T4aN2 disease as stage IIIB, and to classify T4bN2 and T4aN3a as stage IIIC. After we made each of the above adjustments to the staging standards, the differences between stages IIIB and IIIC were not statistically significant (*P* = .438, .681, and .516, respectively) (Fig. 3).

However, when we made adjustments to these 3 standards simultaneously, the survival difference between stage IIIB (n = 221) and stage IIIC (n = 172) patients became larger and reached statistical significance (*P* = .03) (Fig. 2B). Univariable analysis revealed that the HR of stage IIIB before adjustment was higher than that of stage IIIC, at 31.595 (19.915–50.127) and 27.467 (16.785–44.947), respectively; after adjustment, the HR of stage IIIB was lower than that of stage IIIC, at 26.564 (16.685–42.293) and 34.382 (21.502–54.977), respectively.

## 4 Discussion

We previously conducted a statistical analysis of the applicability of the 7th edition UICC TNM gastric cancer classification for Chinese patients according to the 7th edition classification standards released in 2010.^[5]^ We found that it required improvement for late-stage gastric cancer patients, especially in distinguishing the survival rate between stage IIIA and stage IIIB disease. We consulted Japanese gastric cancer classification standards and moved stage T4bN0 from stage IIIB to stage IIIA, and found that this improvement can effectively show the difference in survival rate between stage IIIA and stage IIIB disease.^[6]^ For the patients in the present study, the improved method can still significantly expand the difference in survival between patients with stage IIIA and stage IIIB disease. The improved method also successfully predicted the update to the classification of stage IIIA in the 8th edition. However, the 7th edition classification standards cannot clarify the difference in survival between stage IIIC and stage IV disease, and no significant difference was apparent after the improvement.

Updates involving the N stage have been ongoing. In the most recent 3 decades, both the Japanese TNM classification of gastric cancer and the UICC TNM classification of gastric cancer have undergone several major changes.^[5]^ The 2 standards were different in the classification of the N stage until 2010, when the UICC released the 7th edition of TNM classifications of gastric cancer.^[7]^ Here, we used the 8th edition of UICC TNM classification of gastric cancer as staging criteria for all patients and examined its applicability to Chinese patients. We performed statistical analysis of patient data after the new update. Similar to our previous study, the 8th edition classification also distinguished the survival rate difference between stage IIIA and stage IIIB disease, and also highlighted the difference in survival between stage IIIC and stage IV disease. However, a new problem also emerged, namely a problem in distinguishing the survival rate between stage IIIB and stage IIIC disease. Similar to the problem with the 7th edition classification, the UICC classification has disadvantages in distinguishing the survival rate in Chinese late-stage gastric cancer patients. Furthermore, in the 8th edition classification, the problem in distinguishing survival rate between stage IIIB and stage IIIC patients is even worse. Because the survival curves of patients with these 2 stages of disease are so intertwined, they may even not be separate trends. There can only be a significant difference in the survival curves of stage IIIB and stage IIIC disease if the above 3 improvements are implemented simultaneously.

As the 8th edition is valid for U.S. populations, showing clear separation of data with preservation of group order,^[8]^ we believe that the reasons for this situation include the comprehensive update to the classification standards of late-stage gastric cancer patients in the 8th edition TNM classification and the introduction of the N3b stage. We also admit that the difference between the results of our study and current staging criteria may be partly due to bias in the retrospective patient data. We need to reassess the validity of the staging criteria in patients undergoing surgery in order to obtain more accurate results. More meticulous classification standards are the premise and basis for even more accurate treatment. However, because there are many comprehensive treatment procedures for gastric cancer and their effects on prognosis are difficult to evaluate, the refinement of the classification in a short period of time can increase the risk of confusion between stages. Likewise, we also need to investigate the boundary between stage N3a and N3b in a more scientific manner. In our cohort, there were no stage T1N3b patients. Among stage T1 patients, 7 had the maximum number of lymph node metastases.

Since 2000, there has been a significant increase in the 5-year survival rate of patients with gastric cancer, which may be closely associated with factors such as increased physical examination, screening, and improvements in comprehensive treatment.^[6,9]^ However, the majority of Chinese gastric cancer patients are elderly and from rural areas, which means that the disease is often detected at a later stage. Furthermore, the lack of timely and standardized treatments, as well as poor compliance, are still major issues.^[10]^ From the perspective of applicability of the 7th and 8th edition classification standards for Chinese patients, the main problem still lies in the accurate staging of late-stage patients. Therefore, increasing the rate of early diagnosis and the level of comprehensive treatment of late-stage gastric cancer are important steps for increasing the overall survival rate. We believe that with screening and physical examination becoming increasingly common, more patients could be identified at early and intermediate disease stages.^[10]^

Statistical analysis showed that many factors, including tumor location, Borrmann classification, type of surgery, joint organ removal, and adjunctive therapy can affect the prognoses of patients with gastric cancer. In clinical practice, all of these factors should be considered for gastric cancer classification.^[11–14]^

In our study, some comprehensive treatment information was hard to obtain, and all patient information was obtained retrospectively, leading to a lower reliability of the data than that of clinical trials. Thus, our discussion of categorizing T4aN2 as stage IIIB disease, and T4bN2 and T4aN3a as stage IIIC disease in the 8th edition UICC TNM classification of gastric cancer is for reference only.

## Author contributions

Huizi Gu, Liang Liu, and Hao Zhang participated in the design of the study. Min Li finished the follow-up. Ying Yu and Huizi Gu performed the statistical analysis. Haitao Zhu, Yuxi Wang, and Yu Bai drafted the manuscript. Ping Lu participated in the coordination of the study. All authors read and approved the final manuscript.

**Conceptualization:** Liang Liu, Huizi Gu.

**Data curation:** Ying Yu.

**Formal analysis:** Ying Yu.

**Investigation:** Yu Bai, Min Li.

**Methodology:** Ping Lu.

**Resources:** Haitao Zhu.

**Writing – original draft:** Liang Liu.

**Writing – review & editing:** Yuxi Wang, Hao Zhang.
